# Quality of the calcium-enriched mixture apical plug in simulated apexification model; Effect of different mixing and placement methods

**DOI:** 10.4317/jced.53236

**Published:** 2017-10-01

**Authors:** Saeed Rahimi, Negin Ghasemi, Tahmineh Razi, Akbar Rezaiepour

**Affiliations:** 1 Professor, Dental and Periodontal Research Center, Department of Endodontics, Dental Faculty, Tabriz University (Medical Sciences), Tabriz, Iran; 2 Assistant Professor, Dental and Periodontal Research Center, Department of Endodontics, Dental Faculty, Tabriz University (Medical Sciences), Tabriz, Iran; 3 Assistant Professor, Department of Radiology, Dental Faculty, Tabriz University (Medical Sciences), Tabriz, Iran; 4 Private practice, Tabriz, Iran

## Abstract

**Background:**

Presence of voids at root canal wall‒apical seal material interface gives rise to the entrapment of toxins and microorganisms, which might have a relationship with post treatment disease. The present study was carried out to evaluate the effect of different mixing (manual and ultrasonic) and placement (manual and manual in association with indirect ultrasonic) methods of Calcium-enriched Mixture (CEM) cement on the number and dimensions of voids in the apical plug in simulated apexification models.

**Material and Methods:**

A total of 80 human maxillary central incisors with mature apices were selected. After simulation of the open apices, the teeth were divided into 4 groups (n=20) based on the mixing and placement techniques of CEM cement: group 1, manual mixing‒manual placement; group 2, manual mixing‒manual placement in association with indirect ultrasonic technique; group 3, ultrasonic mixing‒manual placement; and group 4, ultrasonic mixing‒manual placement in association with indirect ultrasonic technique. The samples were placed within gypsum sockets in which the periodontal ligament was reconstructed with polyether impression material. After placement the apical plugs, a wet piece of cotton was placed on canal orifices, followed by dressing with Cavit. The samples were incubated at 37°C and 100% relative humidity for 7 days. Then the voids between the material and root canal walls were counted with the CBCT technique. The void dimensions were scored with the following scoring system: score 1, no voids; score 2, the void size less than half of the size of the evaluated cross-section; score 3, the void size larger than half of the size of the evaluated cross-section. Statistical analyses were carried out with chi-squared and Fisher’s exact tests. Statistical significance was defined at *P*<0.05.

**Results:**

The maximum (7) and minimum (2) number of voids were detected in groups 1 and 2, respectively. The difference between these two groups was statistically significant (*p*<0.05). The differences in the number of voids between groups with similar mixing technique and different mixing techniques (i.e. groups 1 and 3 and groups 2 and 4) were not significant (*p*>0.05). Void dimensions in all the study groups were in score 2 category and no score 3 was recorded in the study groups.

**Conclusions:**

Under the limitations of the present study, manual placement in association with indirect ultrasonic technique was a proper technique to improve the quality of apical plug, considering the decrease in the number of voids.

** Key words:**Apical plug, CEM cement, void.

## Introduction

Calcium-enriched Mixture (CEM) cement is a biomaterial, consisting of different calcium compounds; it has several applications in endodontics and is used as an apical plug in the apexification of mature permanent teeth (1,2). A material applied as an apical plug should provide a proper seal (3) in addition to properties such as setting in the presence of blood and moisture, tissue tolerance and antimicrobial activity (4). It is necessary for such a material to have proper adaptation with the root canal walls to prevent bacterial penetration and leakage of their products into the periapical tissues. Presence of voids at root canal wall‒apical seal material interface gives rise to the entrapment of toxins and microorganisms, which might have a relationship with post treatment disease (5-7).

The careful delivery of a material used as apical plug is very important clinically, considering the limitations in relation to the accessibility and visualization. One of the techniques suggested to create a denser obturation is the use of ultrasonic vibration energy (5,7). Studies on Mineral Trioxide Aggregate (MTA) have shown an increase the density of obturations with the use of indirect ultrasonic energy (7). However, direct use of an ultrasonic tip to place MTA resulted in an increase in the number of surface bubbles (5). No similar studies have been carried out in relation to CEM.

Powder-to-liquid ratio and porosity might affect the mechanical properties of cements used in dentistry. Therefore, the mixing and placement techniques are key factors affecting the performance of dental materials (8).

Evaluation of previous investigations into the effect of mixing technique on various properties of CEM showed that mixing with an ultrasonic tip increases its flow and working time; however, it decreases the setting time, compressive strength and film thickness (9,10). Mixing of the material with the ultrasonic technique does not result in a significant change in bacterial leakage and pH, and the dimensional changes exhibit expansion, with the solubility remaining at an acceptable level (11,12).

Identification of voids is important clinically and evaluation of voids at root canal wall‒material interfaces is a well-established technique for the evaluation of the quality of obturation (6,13). Radiographic techniques are used for such evaluations; however, analog and digital radiographic techniques have some shortcomings because they provide two-dimensional images of 3D structures, with superimposition of anatomic structures (14). In recent years, cone-beam computed tomography (CBCT) technique, which is a non-invasive technique, has been used for the evaluation of dentoalveolar structures (15). This technique employs a low radiation dose, with high resolution. On the other hand, it provides a 3D image, with no geometric distortion (16).

There is no study available on simultaneous evaluation of the effects of various mixing and placement techniques on the quality of CEM apical plugs. This study was therefore undertaken to measure the voids in CEM apical plugs with the CBCT technique by considering the parameters mentioned above.

## Material and Methods

Eighty human maxillary central incisors with straight and mature roots were selected. The tooth samples were free of cracks and fractures. The tooth crowns were cut away at CEJ using a diamond saw (SP 1600 Microtome, Leica, Nu Block, Germany) under water spray to achieve 12 mm of the root length. Then 3 mm of the root was removed from the apical end to leave 9 mm of the root length in all the tooth samples.

-Simulation of periodontal ligament 

The roots were coated with molten wax, followed by mounting in gypsum blocks. After the gypsum (Moldano blue™, Heraues Kulzer, Hanau, Germany) set completely, the wax was eliminated with hot water, which gave rise to a gypsum socket. The socket was filled with polyether impression material (Impregum Soft, 3M ESPE, Seefeld, Germany) and the roots were placed within it. After the polyether impression material set, the roots were removed from the sockets.

-Root canal preparation and simulation of open apex model

The root canals were instrumented with #5, #4, #3, #2 and #1 Gates-Glidden drills (Dentsply Maillefer, Ballaigues, Switzerland) using the crown-down technique in order to simulate apexification. To this end, #5, #4, #3 and #2 drills were inserted into the root canals up to 3, 5, 7 and 9 mm, respectively. To create an open apex, #1 drill was moved 1 mm through the apical foramen once without any resistance. Then the entire length of a #40 RaCe rotary file (FKG, Lachaux-de-Fonds, Switzerland) with 10% taper was inserted into the root canal through the apical foramen. A total of 5 mL of 0.5% NaOCl solution was used for irrigation during instrumentation. At the end of instrumentation, the smear layer was removed with 5 mL of 17% EDTA. A total of 5 mL of normal saline solution was used for final irrigation.

-Placement of the apical plug

The samples were randomly assigned to 4 groups (n=20) in terms of the CEM mixing and placement techniques as follows:

Group 1: manual mixing‒manual placement

Group 2: manual mixing‒manual placement in association with indirect ultrasonic 

Group 3: ultrasonic mixing‒manual placement

Group 4: ultrasonic mixing‒manual placement in association with indirect ultrasonic 

In all the study groups, CEM was mixed following manufacturer’s instructions at a powder-to-liquid ratio of 3:1, with a mixing time of 15 seconds, to achieve a thick consistency. In group 1, subsequent to mixing the material, an MTA carrier was used to transfer the material incrementally into the root canal; the material was condensed with a hand plugger up to the desired length and continued until the apical plug measured 3 mm in length. In group 2, a similar technique was used; however, after condensing the material with a hand plugger an ultrasonic tip was placed in contact with the plugger for 2 seconds so that an indirect vibratory motion was applied to the material. The lowest power settings of the ultrasonic unit were used. In groups 3 and 4, the material was mixed with the ultrasonic tip for 5 seconds; then in group 3, similar to group 1, and in group 4, similar to group 2, the material was placed in the root canal. A wet piece of cotton was placed at root canal orifices and the samples were dressed with Cavit. Then the samples underwent incubation at 37°C and 100% relative humidity for 1 week.

-Evaluation of the samples with the CBCT technique

Each root sample underwent CBCT examination with the use of a Newtom VGI unit (Verona QR, Italy) at axial and cross-sectional views by an experienced radiologist. This CBCT unit delivers a conical x-ray beam and has a flat-panel detector, with 1536×1920 and 127×127 pixel sizes, a pixel depth of 14 bits, a rotation of 360°, a scan time of 18 seconds and a kVp of 110. NNT Viewer software program version 2.17 carried out the initial and final reconstructions. The exposure conditions were set automatically. Data collected from the CBCT examinations were entered into the NNT Viewer software program version 2.17. The images were displayed on a 19-inch LCD monitor (PHILIPS, 190B) with a resolution of 1024×1208 pixels and 32 bits in a dimlylit room. The number of voids at material‒root canal wall interfaces was counted in all the samples and recorded in terms of the number of voids in 20 samples in each group. The following scoring system was used to characterize the dimensions of the voids (5):

Score 1: no voids

Score 2: the size of the void less than half the size of the cross-section evaluated

Score 3: the size of the void larger than half of the size of the cross-section evaluated

-Statistical analysis

After drawing cross tabs for the data, statistical analyses were carried out with chi-squared and Fisher’s exact tests.

## Results

 Table 1 presents the number and dimensions of the voids in the study groups. The maximum (7) and minimum (2) number of voids were detected in groups 1 and 2, respectively. The difference between these two groups was significant (*P*=0.001). Groups 1 and 3 and groups 2 and 4 did not exhibit any significant differences in the number of voids (*p*>0.05). Evaluation of the dimension of the voids showed no score 3 in any of the study groups and the dimensions of all the bubbles conformed to score 2, ( Table 1).

**Table 1:**
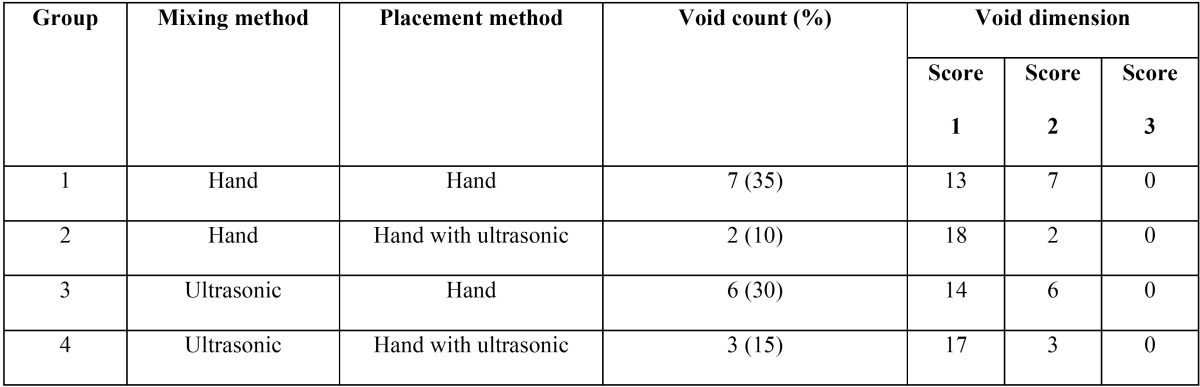
Void counts and dimensions in the study groups.

## Discussion

The present study was undertaken to evaluate the effect of different mixing and placement techniques of CEM cement on the number and dimensions of voids in the apical plug. The results showed the positive and significant effect placement of the plug with indirect application of ultrasonic technique in association with the manual technique on decreasing the number of voids in the apical plug. However, the mixing technique had no significant effect on the quality of the apical plug. In other words, in groups with identical placement technique, there were no significant differences in the number and dimension of voids with hand and ultrasonic mixing techniques.

Placement of an apical plug in a tooth with an open apex is a technique-sensitive process due to the absence of an apical stop and limitations in visibility and access and it is always possible for voids to form between the apical plug and the root canal walls. These voids provide a path for leakage and create a space for proliferation of microorganisms, compromising the seal and paving the way for post treatment disease (4,6,17).

In the present study, the number and dimension of voids in the apical plug between the material and the root canal wall were evaluated with the use of the CBCT technique in the axial and cross-sectional vies at 0.1-mm accuracy. In two similar previous studies on the quality of MTA obturation, light microscopy, radiography (5) and weighing of the samples (7) were used to determine the density of obturation. The method used in the first study was not possible in our study because in that study polyethylene tubes were used as root canals, which were eliminated with heat after obturation and then the samples were evaluated. In the present study, natural teeth were used to more closely simulate the clinical conditions. The second technique, i.e. weighing of the samples, did not consider the surface voids of the material, which is one of the limitations of the mentioned study.

A method suggested for improving the density and homogeneity of the apical plug is to use the vibration energy during placement and packing of the material, which re-arranges the particles and creates a denser mass by creating compressive impulses (7). A previous study by Yeung et al has substantiated this (7). The similarity between the results of that study and the present study was the positive effect of indirect ultrasonic energy in association with manual condensation of the material. Of course the study designs of the two studies are different and it is not possible to directly compare the results of the two studies. The main differences between these two studies, in a addition to the evaluation technique consisting of weighing the samples in that study, consisted of the shorter duration of application of the ultrasound (1 second), use of acrylic block canals which simulated a closed apex, and combination of the mixing technique with the placement technique. In contrast, in the present study natural teeth with a standard model of simulated open apex was used with placement of samples within a socket with simulated periodontal ligament. Condensation of an apical plug material in a tooth with open apex is much more difficult than that in a tooth with closed apex. The ultrasonic energy in the present study was applied for 5 seconds.

The results of the present study are in contrast to those of a study by Aminshariae *et al.* (5), in which the direct application of ultrasonic energy resulted in an increase voids on the surface of the MTA. The difference between the present study and that study was the shorter time and the technique used to apply the ultrasonic energy; in that study the ultrasonic energy was applied for 30 seconds, which is longer than that in the present study. Previous studies have shown that longer application of ultrasonic energy and a higher energy level increase void formation (7). In the present study, the power setting of the ultrasonic unit was adjusted at the minimum and the ultrasonic energy was applied indirectly because even the thinnest ultrasonic tips reach the root canal apical end with difficulty in the clinic. With the direct application of the ultrasound energy, the odds of void formation are high due to the direct induction of vibration.

One of the differences between the present study and the two studies mentioned above was the apical plug material. In the present study, CEM was used but in the studies mentioned above, MTA was used. Comparison of the properties of these two materials in previous studies has shown the easier handling and higher flow of CEM compared to MTA (2,18).

Apart from the placement technique, another parameter evaluated in the present study, which was not taken into account in previous studies was the mixing techniques of the material before its placement.

Previous studies have shown the effect of mixing technique on a number of physical properties of biomaterials such as CEM (9-12,18,19). In the present study, there were no differences in the number and size of voids between the two groups that had similar placement technique; in other words, the effect of the mixing technique was not significant in contrast to the placement technique. In groups in which ultrasonic placement was used in association with the manual technique, manual and ultrasonic mixing had a similar effect on the dependent variables of the study; however, in previous studies (9-12,18,19) in which the other properties of CEM were evaluated with different mixing techniques, it was shown that ultrasonic mixing resulted in a decrease in setting time and an increase in the material’s flow, with no unfavorable effect on the properties such as the sealing ability. Therefore, use of this mixing technique is suggested to benefit from its positive effects.

One of the limitations of the present study was the absence of curved canals. It is suggested that curved canals be used in future studies. In previous studies, the effect of indirect ultrasonic placement technique on increasing the density of the obturation was more evident in curved canals compared to straight canals (7).

## Conclusions

Under the limitations of the present study, it can be concluded that when CEM is used as an apical plug, it is recommended that ultrasonic mixing and placement with manual condensation in association with indirect ultrasonic energy be considered in a short time and with minimum power.
